# Peculiarities of Oxidative Polymerization of Diarylaminodichlorobenzoquinones

**DOI:** 10.3390/polym13213657

**Published:** 2021-10-23

**Authors:** Andrey V. Orlov, Svetlana G. Kiseleva, Galina P. Karpacheva, Dmitriy G. Muratov

**Affiliations:** A.V. Topchiev Institute of Petrochemical Synthesis, Russian Academy of Sciences, Leninsky pr., 29, 119991 Moscow, Russia; avorlov@ips.ac.ru (A.V.O.); skisel@ips.ac.ru (S.G.K.); muratov@ips.ac.ru (D.G.M.)

**Keywords:** oxidative polymerization, diarylaminodichlorobenzoquinones, polyaniline

## Abstract

New oxidative polymerization monomers—diarylaminodichlorobenzoquinones were synthesised by alkylating aniline, m-phenylenediamine and methanilic acid with chloranil. Oxidative polymerization of diarylaminodichlorobenzoquinones was studied for the first time in relation to the concentration of the monomer, acid, and oxidant/monomer ratio. It was found that the synthesized monomers are highly active in the polymerization reaction, and the oxidation rate grows with the increase in the acid concentration. Only one arylamine group is involved in the polymerization reaction. The optimal oxidant/monomer ratio is stoichiometric for one arylamine group, despite the bifunctionality of the monomers. It was shown that the type of the substituent in the aniline ring (electron donor or electron acceptor) determines the growth of the polymer chain and the structure of the resulting conjugated polymers. A mechanism for the formation of active polymerization centers for diarylaminodichlorobenzoquinones was proposed. FTIR-, NMR-, X-ray photoelectron spectroscopy, and SEM were used to identify the structure of the synthesized monomers and polymers. The obtained polymers have an amorphous structure and a loose globular morphology. The frequency dependence of the electrical conductivity was studied.

## 1. Introduction

Currently, the rapidly growing industry of materials for various fields of electronics and electrical engineering is at the stage where it requires new types of conductive and electroactive polymers with a set of certain properties. To create such polymers, serious fundamental and applied problems should be solved, associated with the development of methods for obtaining stable technologically acceptable materials characterized by high-level functional parameters. Conductive polymers with a system of conjugated double bonds hold a special place. The electronic structure of conjugated polymers, associated with the delocalization of π-electrons along the conjugation chain, determine their excellent electrophysical, optical, electrochemical, membrane, and other properties [1,2,3,4,5,6,7,8,9,10,11].

Polyaniline (PANI) is the oldest and the most common representative of conducting polymers due to its easy synthesis and doping-dedoping processes, low cost, high electrical conductivity, good redox properties, environmental stability, etc. [11,12,13,14,15,16,17,18,19,20,21,22,23,24,25]. However, the rigidity of the polymer chain and, consequently, its inability to melt and insolubility in most organic solvents limit the potential application of PANI in modern technologies. Much effort has been put into the study of the oxidative polymerization of its derivatives, aiming to obtain easily processed products with improved properties. Yet, compared to aniline, its C-, N-derivatives were either inactive in the oxidative polymerization reaction, or resulted in low molecular weight products with poor electrophysical characteristics [26,27,28,29]. The study of the oxidation reaction of C-substituted anilines showed that ortho- and meta-halogen-substituted aniline form low molecular weight products (M_w_ = 1–3 × 10^3^ g/mol) with low electrical conductivity (1 × 10^−4^–10^−10^ Scm^−1^). Sulfonated derivatives of aniline, regardless of the position of the substituent in the ring, form soluble oligomers in low yields with an electrical conductivity not exceeding 1 × 10^−2^ S⋅cm^−1^ [30,31,32]. Attempts to synthesize polymers of nitro- and cyan-derivatives of aniline have not been successful. Numerous studies have shown that the oxidation rate of monomers with electron donor substituents is higher than that of monomers with electron acceptor substituents. The yield and molecular weight of polymers depend on the volume of the substituent and its position in the ring. For example, for ortho-methyl-, ortho-ethyl-, ortho-propylaniline, the yield of polymers drops from 80% to 2%. A substituent in the meta-position reduces the oxidation rate even more. The molecular weight of the obtained polymers is within the range of 1–7 × 10^3^ g/mol [30,31,32,33]. Methoxyaniline with a substituent in the meta-position is not active in the oxidative polymerization reaction. When a substituent is in the ortho-position, this monomer gives a polymer with a molecular weight of 2 × 10^3^ g/mol and electrical conductivity up to 10^−3^ S⋅cm^−1^. Amino and hydroxy groups in the aromatic ring activate the oxidative polymerization, but, during the oxidation, phenylenediamines and aminophenols themselves form either low molecular weight soluble products or crosslinked polycyclic structures [28,34,35,36,37,38,39]. The electrical conductivity of polyphenylenediamines was in the range of 10^−4^–10^−6^ S⋅cm^−1^ [34,36]. It turned out to be impossible to polymerize the monomers with aryl substituents in the ortho- and meta-position of the benzene ring. At the same time, polymerization of monomers with aryl substituents in the para-position quite easily leads to the formation of polymers containing biphenylene units with the electrical conductivity below 10^−4^ S⋅cm^−1^ [40,41].

As for N-substituted anilines, the oxidative polymerization of alkyl and aryl derivatives has been studied. N-alkylanilines polymerization produces easily hydrolysable products of low molecular weight [42]. The electrical conductivity depends on the volume of the alkyl substituent and increases from 10^−4^ S⋅cm^−1^ for polybutylaniline to 10^-2^ S⋅cm^−1^ for polymethylaniline. Polymerization of N-aryl derivatives of aniline leads to the formation of low molecular weight products of benzidine structure with electrical conductivity below 10^−4^ S⋅cm^−1^ [40].

The alkylation reaction of aniline and other aromatic amines remains advantageous and easily available for obtaining N-substituted aniline derivatives. For example, alkylation of aniline and its derivatives with chloranil followed by cyclization is used to produce triphenoxazine dyes. Various diarylaminodichlorobenzoquinones have been obtained via alkylation of aromatic amines with chloranil [43]. However, until now, these compounds have not been considered as monomers for oxidative polymerization reaction.

In this article we discuss the results obtained for the first time of a study on the oxidative polymerization of new diarylaminodichlorobenzoquinone monomers, such as: 2,5-dianiline-3,6-dichloro-1,4-benzoquinone, 2,5-diphenylenediamino-3,6-dichloro-1,4-benzoquinone, 2,5-di(aniline-3-sulfo)-3,6-dichloro-1,4-benzoquinone, 2-(aniline-3-sulfo)-5-anilino-3,6-dichloro-1,4-benzoquinone as well as 2-methoxy-5-anilino-3,6-dichloro-1,4-benzoquinone. All synthesized monomers have a strong electron acceptor substituent in their structure—a bulky chloroquinone group at the nitrogen atom, which should lead to a decrease in the electron density at the nitrogen atom. Together with the steric factor, this should have led to a sharp decrease in the activity of such monomers in the oxidation reaction in comparison with aniline. However, the synthesized monomers demonstrated high activity during oxidative polymerization. Features of the oxidative polymerization of the obtained monomers were studied in comparison with the polymerization of aniline and depending on their structure and reaction conditions. A formation mechanism for active polymerization centers was proposed, explaining their high activity in the oxidation reaction. The chemical structure, morphology and electrical properties of the obtained new conjugated polymers were studied.

## 2. Experimental

### 2.1. Materials

Chloranil (CA) (reagent grade) was recrystallized from dioxane; aniline (reagent grade) was distilled twice at the residual pressure of 1.33 kPa and the temperature of 50 °C; metanilic acid (MA) (reagent grade) was recrystallized from water; dioxane (reagent grade) was distilled at 101 °C; bidistilled water was used. Ammonium peroxydisulfate (reagent grade) (APS) was recrystallized from water at T = 40 °C. Methanol (extra pure grade), sodium acetate (extra pure grade), ethanol (extra pure grade), HCl solutions (analytical grade), NH_4_OH (extra pure grade), m-phenylenediamine (m-PDA) (98%, Acros Organic, Belgium) were used without additional purification.

### 2.2. Synthesis of Monomers

#### 2.2.1. Synthesis of 2,5-Dianiline-3,6-dichloro-1,4-benzoquinone (DADCB)

DACB was obtained via alkylation of aniline with CA. A solution of 0.05 mol of CA in 200 mL of dioxane was added dropwise to a solution of 0.1 mol of aniline in 50 mL of dioxane during an hour under cooling (15 °C) and constant stirring. After the reagents were mixed, the reaction solution was stirred at room temperature for another 3 h. Then, the reaction mass was filtered, the red-brown precipitate was washed with ethanol and dried under vacuum at room temperature to constant weight. The yield of DADCB was 92% of the yield predicted by theory. Elemental analysis: C—60.2%; H—3.4%; N—7.8%; O—8.9%. ^1^H NMR: δ_NH_ 8.3 ppm; δ_PhH_ 7.1–7.4 ppm. The monomer is highly soluble in DMFA, DMSO, N-MP and poorly soluble in dilute acid solutions.

#### 2.2.2. Synthesis of 2-Methoxy-5-anilino-3,6-dichloro-1,4-benzoquinone (MADCB)

MADCB was obtained via two-step synthesis. At the first stage, 2-methoxy-3,5,6-trichloro-1,4-benzoquinone (MTCB) was obtained via the reaction of CA and methyl alcohol in the presence of sodium acetate. A total of 0.02 mol of sodium acetate were dissolved in 200 mL of methanol, then 0.02 mol of CA were added. The reaction continued for 4 h at heating the reaction mixture to methanol boiling point. The color of the reaction solution changed from yellow to red-brown, then a precipitate formed. After the reaction was complete, the reaction mixture was filtered. The resulting red-orange crystalline fine powder was washed with cold methanol to a colorless solution and dried under vacuum at room temperature to constant weight. The MTCB yield was ~ 68% of the weight predicted by theory. MTCB dissolves in ethyl alcohol, dioxane, DMSO, DMFA. Elemental analysis: C—34.93%, H—1.45%, O—19.07%. ^1^H-NMR: δ_H/OCH3_ 3.48 ppm; δ_PhH_ 7.08, 7.15, 7.39 ppm.

MADCB was synthesized via the reaction of MTCB and aniline in ethyl alcohol. A solution of 0.02 mol of aniline in 30 mL of alcohol was added dropwise to a solution of 0.02 mol of MTCB in 220 mL of ethanol for 10 min at cooling to 15 °C. After the reagents were mixed, the reaction system was stirred at room temperature for another 4 h. The resulting golden greenish-brown fine powder was filtered off, washed with cold alcohol, and dried under vacuum. MADCB dissolves in dioxane, DMSO, DMFA and poorly soluble in dilute acid solutions. The yield was 63%. Elemental analysis: C—52%, H—3.52%, N—4.48%, O—16%. ^1^H-NMR: δ_H/Ph_ 7.08, 7.15, 7.39 ppm; δ_H/OCH3_ 3.52 ppm.

#### 2.2.3. Synthesis of 2,5-Diphenylenediamino-3,6-dichloro-1,4-benzoquinone (DPDCB)

DPDCB was obtained via alkylation of m-PDA with CA. A solution of 0.05 mol of CA in 200 mL of dioxane was added dropwise to a solution of 0.1 mol of m-PDA in 50 mL of dioxane during one hour at room temperature and under constant stirring. After the reagents were mixed, the reaction solution was stirred for another 3 h at room temperature. Then, the reaction mass was filtered, the brown powder was washed with ethanol and dried under vacuum at room temperature to constant weight. The yield of DPDCB was 78% of the yield predicted by theory. Elemental analysis: C—55.2%; H—3.6%; N—14.6%; O—8.2%. ^1^H-NMR: δ_NH_ 8.4; δ_H/Ph_ 6.61, 6.82, 7.15 ppm. The monomer is soluble in DMFA, DMSO, N-MP and in aqueous solutions of acids.

#### 2.2.4. Synthesis of 2,5-Di(aniline-3-sulfo)-3,6-dichloro-1,4-benzoquinone (DASDCB)

DASDCB was obtained via the reaction of CA and MA in ethyl alcohol. A total of 30 mL of alcohol solution of MA (0.02 mol) and sodium acetate (0.08 mol) were added to a solution of CA (0.01 mol) in 100 mL of ethanol. The reaction continued for 4 h at 30 °C. The obtained light-brown fine powder is highly soluble in aqueous solutions of acids and partially soluble in water and alcohol. The product yield is ~72%. Elemental analysis: C—42%, H—2.93%, N—5.99%, O—23%, S—12.08%. ^1^H-NMR: δ_H/Ph_ 7.14, 7.39, 7.53 ppm; δ_H/PhSO3H_ 11.8 ppm. The monomer is soluble in DMFA, DMSO, N-MP and in aqueous solutions of acids.

#### 2.2.5. Synthesis of 2-(Aniline-3-sulfo)-5-anilino-3,6-dichloro-1,4-benzoquinone (ASADCB)

ASADCB was obtained via two-step synthesis. At the first stage, 2-(aniline-3-sulfo)-3,5,6-trichloro-1,4-benzoquinone (ASTCB) was synthesized via alkylation of MA with CA in dioxane. 60 mL of an alcohol solution of MA (0.01 mol) and sodium acetate (0.01 mol) were added dropwise to a solution of 0.02 mol of CA in 200 mL of ethyl alcohol during 30 min under constant stirring. After the reagents were mixed, the reaction solution was stirred for another 4 h at T = 40 °C. Then, the mixture was cooled to room temperature, the reaction mass was filtered, the precipitate was washed with ethanol and dried under vacuum at room temperature to constant weight. The ASTCB yield was ~60% wt. of the yield predicted by theory. ASTCB dissolves in dioxane, DMSO, DMFA and poorly soluble in dilute acid solutions. Elemental analysis: C—34.93%, H—1.45%, N—4.63%, O—19.07%.

At the second stage, 0.01 mol of ASTCB and 0.015 mol of aniline were mixed in 400 mL of dioxane. The reaction was carried out for 4 h under heating (T = 50 °C) and constant stirring. Then, the reaction mixture was cooled to room temperature and filtered. The light brown powder was washed with ethanol and dried under vacuum at room temperature to constant weight. The yield of ASADCB was 45%. ASADCB dissolves in DMSO and DMFA. Elemental analysis: C—48%, H—3%, N—5.8%, O—18%, S—6.5%. ^1^H-NMR: δ_H/Ph_ 7.2, 7.46, 7.69, 7.9 ppm; δ_H/PhSO3H_ 12.1 ppm.

### 2.3. Oxidative Polymerization of the Obtained Monomers

To better dissolve the synthesized monomers in aqueous solutions of acids, the ultrasonic dispersion was used. Before oxidative polymerization, the monomer solution was stirred using an ultrasonic dispersant MEF 91.1 (600 W) (Russia) for 15 min at 5 °C, then thermostated at the synthesis temperature under constant stirring. The oxidizing agent—APS in the acid solution—was added as a single step. Standard polymerization conditions had the following parameters: [monomer] = 0.03 mol/L; [APS]/[monomer] = 1.25; [HCl] = 0.5 mol/L; T = 20 °C. In cases when the parameters were changed, they were noted additionally in the description of the process. After the reaction was completed, the reaction mixture was filtered, washed with 0.1 M HCl solution and water, and dried under vacuum at room temperature to constant weight. Dedoping was carried out for 3 h with 0.5 M NH_4_OH solution, after that the mixture was filtered, washed with water, and dried under vacuum. Doped polymers are insoluble in organic solvents. In dedoped form, the polymers are soluble in DMFA, DMSO, N-MP. Table S1 shows the yield values and molecular weight characteristics of the obtained polymers.

### 2.4. Characterization

Kinetic studies of the oxidative polymerization of monomers were carried out via the potentiometric method using a 4-channel “Ekspert-001-3(0.4)” ionomer (Russia) with the accuracy of EMF = ± 1.5 mV, registering changes in EMF depending on the reaction time. A redox electrode (ERP-105) (Russia) was used as an electrode.

Fourier transform infrared (FTIR) spectra were recorded in the ATR mode using a HYPERION-2000 IR microscope coupled with a Bruker IFS 66 V/s FTIR spectrometer (Ge crystal, scan 100, resolution 2 cm^−1^, range 600–4000 cm^−1^). Optical density was D = lgI_0_/I.

X-ray photoelectron spectroscopy (XPS) study was performed using a PHI5500 Versa Probe II X-ray photoelectron spectrophotometer. The excitation source was monochromatic Al Kα radiation (hν = 1486.6 eV), power—50 W, diameter—200 μm. Atomic concentrations were determined from the survey spectra (C1s, N1s, O1s, Cl2p lines) using the method of relative sensitivity factors of elements. The bond energies (E_b_) of photoelectron lines (C1s, N1s, O1s, Cl2p) were determined from high-resolution spectra recorded at the analyzer transmission energy of 23.5 eV and the data collection density of 0.1 eV/step. The non-linear least square method using the Gauss-Lorentz function was applied to approximate the spectra. The E_b_ bond energy scale was calibrated at Au4f—84.0 eV and Cu2p3—932.6 eV. The E_b_ scale was corrected according to the binding energy of the N1s spectrum (399.9 eV). The error in determining E_b_ was ±0.2 eV, the error in determining the intensity of the peaks upon approximation was ± 5%.

NMR spectra were recorded on a spectrometer AVANCE III HD at 400 MHz.

The molecular weight of polymers was measured by GPC on a Milton Roy instrument equipped with a Milton Roy RI-detector and a PLgel 5um MIXED-C column using N-MP as an eluent at 60 °C. The eluent flow rate was 1mL min^−1^. The volume of injected sample was 150 μL. Polystyrene was used for the calibration. The accuracy of MW determination was −5%.

SEM photomicrographs were obtained using a scanning electron microscope with an NVision 40 thermal emission source (Carl Zeiss, Oberkochen, Germany). For the study, the samples were applied in the form of a powder onto a conductive carbon tape. The accelerating voltage of the electron gun was 1–20 kV. Images were obtained in secondary electrons at magnifications up to 800,000× and were recorded digitally on a computer.

X-ray diffraction (XRD) analysis was performed using a diffractometer “Difray” 401 (Scientific Instruments, Russia) with Bragg–Brentano focusing, using Cr-Kα radiation at room temperature.

The conductivity σ_ac_ was measured with 6367A precision LCR meter (Microtest Co., New Taipei City, Taiwan) in the frequency range 0.25 Hz–1.0 MHz.

## 3. Results and Discussion

**Scheme 1:**
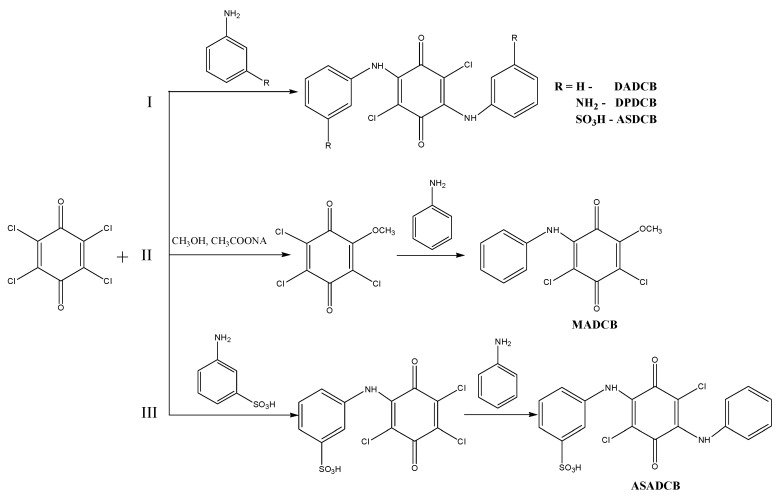
Synthesis of monomers: I—DADCB, DPDCB, ASDCB; II—MADCB; III—ASADCB.

The DADCB, DPDCB, DASDCB monomers were obtained by alkylation of aniline, m-PDA, and MA with CA. Products with a symmetric structure were obtained by a one-step method (Scheme 1I). In their composition, they have two identical structural units of arylamine, potentially active in the oxidation reaction.

Asymmetric MADCB (Scheme 1II) and ASADCB (Scheme 1III) monomers were obtained via a two-step sequential alkylation process.

### 3.1. The Oxidative Polymerization of 2,5-Dianiline-3,6-dichloro-1,4-benzoquinone (DADCB)

**Figure 1:**
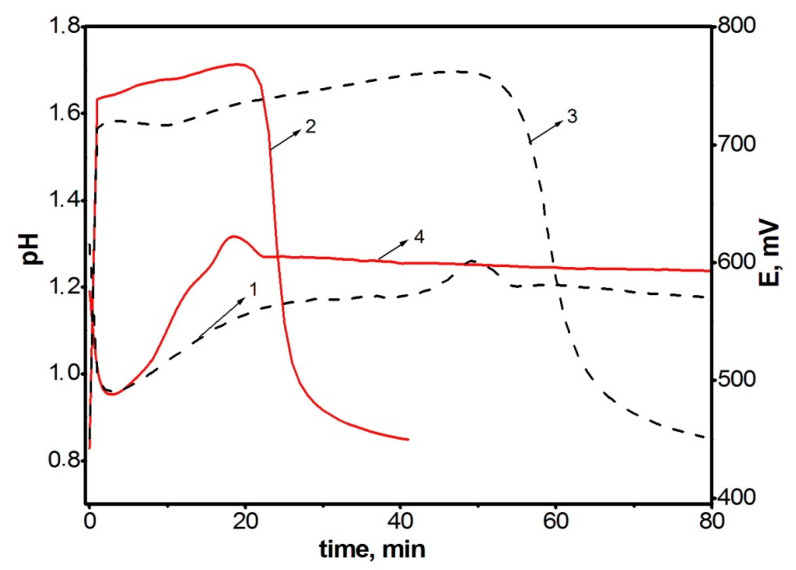
Time dependences of changes in pH (curves 1 and 2) and redox potential (curves 3 and 4) at the oxidation of aniline (curves 1 and 3) and DADCB (curves 2 and 4). Standard conditions.

Since the polymerization reaction of diarylaminodichlorobenzoquinones is a redox process and is researched within low concentration limits of reagents (0.01–0.05 mol/L), and the solutions have an intense color, it seems reasonable to use time dependences of the change in the redox potential of the reaction solution to assess the reaction rate. Figure 1 shows time dependences of pH (1, 2) and redox potential (3, 4) at the oxidation of aniline (1, 3) and DADCB (2, 4). Potentiometric curves have a simple and clear view and correspond to the nature of processes occurring during oxidative polymerization. It can be seen that when the oxidizing agent is added to the monomer solution in the reaction mixture, the equilibrium between the components of the redox reaction is achieved quite soon. Then, after a certain induction period, the redox potential values begin to drop. The time corresponding to the beginning of the decrease in the EMF values on potentiometric curves 3 and 4 corresponds to a decrease in pH on the respective curves 1 and 2, which indicates the oxidative polymerization reaction. In the case of aniline polymerization, this stage is accompanied by precipitation of PANI from the solution and the change of the reaction solution color from dark purple at the beginning of the reaction to dark green by the end of the process.

The first thing that attracts attention is that the oxidation reaction of DADCB proceeds much faster than that of aniline. This is revealed primarily by the shortened induction period. It should be noted that at the beginning of the DADCB oxidation reaction the solution is not of dark purple color, typical for the polymerization of aniline. This is due to the formation of an excess of oxidized quinodiimine fragments involved in autocatalysis and, when the polymerization of aniline is complete, reduced with the formation of the emeraldine form of the polymer [44,45]. During the oxidation of DADCB, the reaction solution darkens gradually, acquiring a dark green color.

It can be assumed that, in contrast to the polymerization of aniline, the presence of pernigraniline structures is not a feature of the polymerization of DADCB. The presence of quinodiimine units is generally not typical for polymers based on N-substituted anilines [46]. The results of FTIR spectroscopy of the poly-DADCB structure confirm this assumption. Figure S1 shows FTIR spectra of the DADCB monomer and the polymer based on it.

**Scheme 2:**
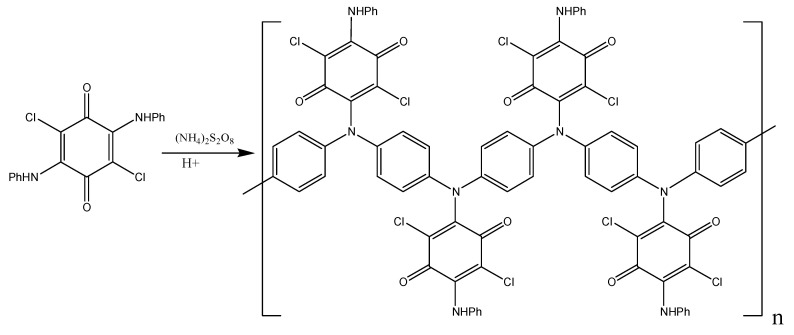
Poly-DADCB synthesis scheme.

As can be seen from Figure S1, both spectra manifest clear absorption bands of N-H bonds at 3231 cm^−1^, two weak bands corresponding to CH bonds in aromatic rings at 3040–3060 cm^−1^. The intensity ratio of these bands in the polymer changes, which is natural, since, apart from monosubstituted rings in the monomer, the polymer also has p-disubstituted rings [43,47,48,49,50]. The bands at 1652, 1005–1059 cm^−1^, and 721–733 cm^−1^ in the spectra of the monomer and polymer belong to the quinoid structure of the chloranil ring [43,47,51]. Intense bands in the range of 1311–1603 cm^−1^ characterize skeletal vibrations in aromatic aniline rings. Significant changes in the polymer spectrum compared to the monomer spectrum occur within the range of deformation vibrations of C–H bonds of aromatic rings (691–850 cm^−1^): there appears a new intense band at 833 cm^−1^ assigned to p-substituted aromatic rings [48,49,50]. Thus, as in the case of aniline polymerization, the oxidative polymerization of DADCB proceeds according to the head-to-tail type with the formation of para-substituted phenyleneamine units (Scheme 2).

In the spectrum of the polymer, the band corresponding to C=C bonds in aromatic aniline rings lies at 1498 cm^−1^, whereas the spectrum of PANI shows this band at 1502 cm^−1^. Quinodiimine structures typical for PANI cause a band at 1590 cm^−1^ [49,50,51]. The spectrum of poly-DADCB has no band at 1590 cm^−1^. The weak band at 1597 cm^−1^ is associated with the chloranil fragment in this polymer [51,52].

It is hard not to notice significant changes in the absorption region of the monosubstituted aromatic ring at 690–760 cm^−1^. The monomer spectrum demonstrated two bands at 986 and 694 cm^−1^ and three bands at 738, 746, and 755 cm^−1^ corresponding to out-of-plane CCH bending vibrations in the monosubstituted ring, whereas the polymer spectrum shows one band at 691 cm^−1^ and two at 746 and 756 cm^−1^ corresponding to the same vibrations [43,47,48,51]. This can be explained by the rather low symmetry of substituted aniline rings in the monomer and an increase in the symmetry of such rings in the polymer, i.e., one may talk about the conformational heterogeneity of aniline groups in the monomer and more one-type conformations at such sites in the polymer. This is due to the fact that bulky substituents located along the PANI chain are superimposed, forming stacks in which their spatial orientation is strictly restricted. As a result, the conformational uniformity in the aniline rings in the polymer is significantly increased.

Thus, the structure of the poly-DADCB chain is similar to the PANI chain. The only difference is the presence of bulky CA substituents with aniline fragments.

To answer why the oxidation of DADCB is faster than the oxidation of aniline and why the second reaction center is not involved in the polymerization reaction, the effect of the reaction conditions on the DADCB oxidation rate was studied.

**Figure 2:**
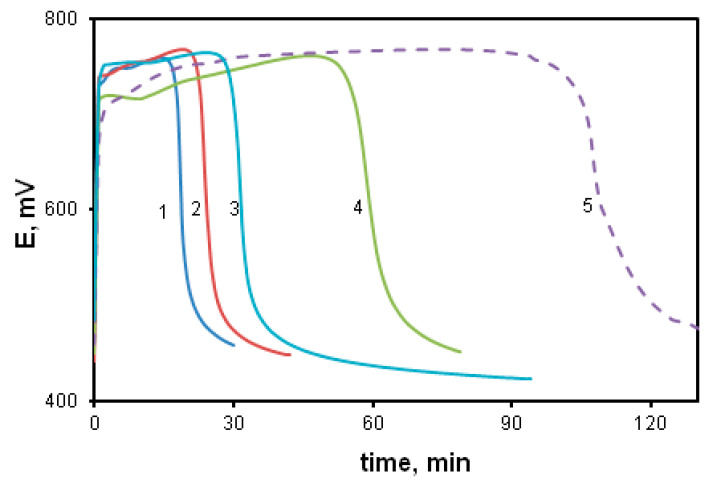
Changes in redox potential with time at the oxidation of DADCB: [DADCB] = 0.05 mol/L (1), 0.03 mol/L (2), 0.02 mol/L (3), 0.005 mol/L (4). (Standard conditions). (5)—polymerization of DACB at T = 0 °C.

The increase in the monomer concentration leads to the acceleration of the oxidative polymerization of DADCB, just as in the case of the polymerization of aniline. Figure 2 shows potentiometric curves of DADCB oxidation depending on the monomer concentration. It can be seen that the increase in the monomer concentration from 0.01 mol/L to 0.05 mol/L (curves 1–4) leads to a significant shortening of the induction period. The process is influenced considerably by the reaction temperature. A decrease in the synthesis temperature to 0 °C leads to an almost five-fold lengthening of the induction period (curve 5).

**Figure 3:**
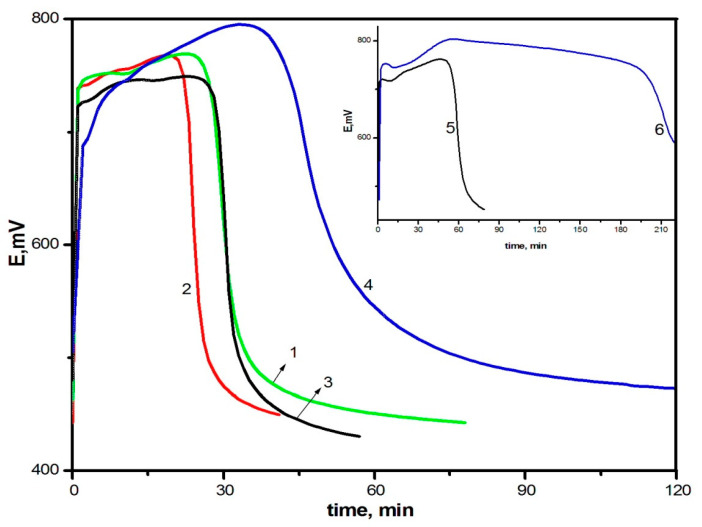
Changes in redox potential with time at the oxidation of DADCB: [Ox]/[DADCB] = 0.75 (1), 1.25 (2), 1.75 (3), 2.5 (4), and at the oxidation of aniline: [Ox]/[An] = 1.25 (5), 2.5 (6).

The study of the effect of oxidant concentration on the polymerization of DACB showed that the increase in the oxidant/monomer ratio from 0.75 to 1.25 (Figure 3, curves 1 and 2) leads to the shortening of the induction period and the increase in speed, as is observed during the polymerization of aniline [53]. However, when the oxidant/monomer ratio is increased to 1.75 and further on to 2.5 (curves 3 and 4, respectively), a sharp increase in the induction period and a decrease in the reaction rate are observed, despite the fact that DACB is a bifunctional monomer and the oxidizer/monomer ratio of 2.5 corresponds to oxidation stoichiometry of its amino groups. A similar picture is observed in the case of aniline oxidation (curves 5, 6). Since the observed effect applies to both aniline and DACB, it can be assumed that this phenomenon is not related to the number of aniline units in the monomer.

**Figure 4:**
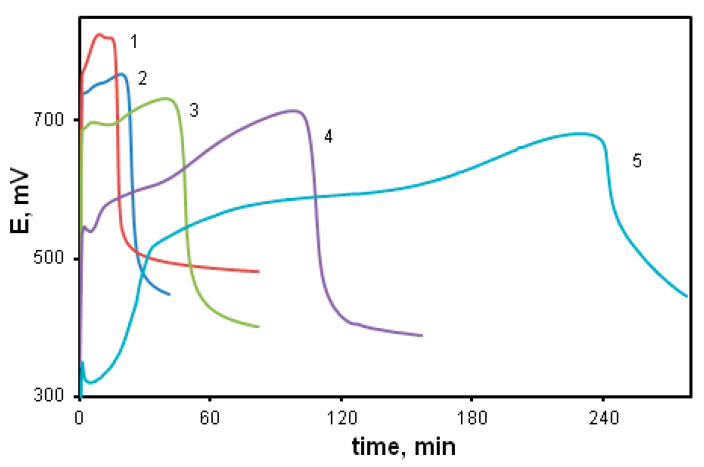
Changes in redox potential with time at the oxidation of DADCB in various media: [HCl] = 0.5 (1), 0.1 (2), 0.02 mol/L (3); water (4); [NH_4_OH] = 0.05 mol/L (5).

A change in acid concentration also leads to significant changes in the kinetics of the process. Figure 4 shows potentiometric curves of DADCB polymerization at various acid concentrations, as well as in neutral and alkaline media. It can be seen from the figure that pH values mostly affect the induction period. The higher the pH value, the lower the reaction rate at the initial stage (curves 1–3). A similar picture is observed in the polymerization of aniline [13,53]. However, when the reaction is carried out under weak acidic and alkaline conditions (curves 4 and 5), the process of DADCB polymerization is somewhat different from the polymerization of aniline, which is characterized by a shortening of the induction period with the increase in the pH of the solution. In addition, during the polymerization of aniline, the kinetic curves acquire a two-step form [13,53], associated by the authors with a change in the oxidation reaction mechanism at high pH of the medium and with a different structure of the resulting intermediates. In our case, the induction period grows with the increase in pH, and the shape of the curves shows hardly any change. Only a slight decrease in the initial redox potential is observed, and the potentiometric curves become more stretched. It is possible that, in the case of DADCB, a change in the reaction medium pH does not alter the oxidation mechanism, but only accelerates or slows down the process.

So, the analysis of the obtained data makes it possible to draw the conclusion that in both cases the concentration of the oxidizing agent exceeding a certain limit leads to the reaction slowdown. However, in the case of DADCB, it does not agree with the stoichiometry of the reaction corresponding to a monomer with two aniline groups. This indicates only one reaction center involved in the polymerization process. An increase in the acid concentration accelerates the process of DADCB oxidation more than an increase in the concentration of the oxidizing agent. Molecular weight of poly-DADCB obtained under standard conditions after dedoping was M_w_ = 2.72 × 10^4^ g/mol.

**Scheme 3:**
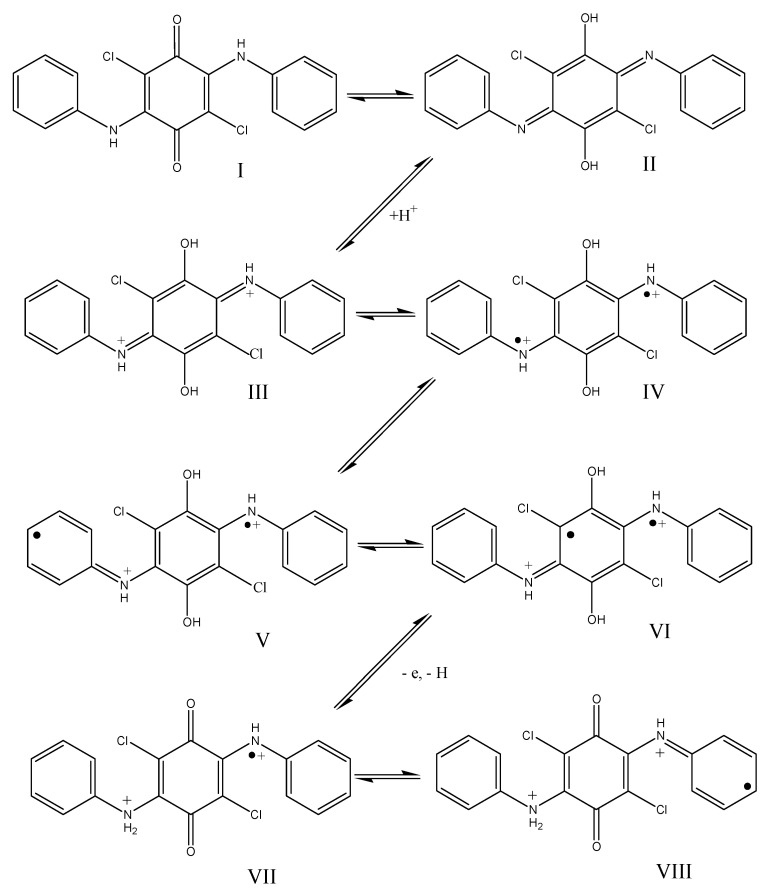
Presumptive mechanism for the formation of active centers during tautomeric rearrangements and oxidation of diarylaminodichlorobenzoquinones. I—quinone; II—quinodiimine; III—dication; IV, V, VI—possible resonance structures, VII, VIII—cation radicals.

To explain the differences in the polymerization of DADCB as compared to the polymerization of aniline, we assumed that a DADCB molecule, which is a tetrasubstituted quinone with two aniline substituents, can undergo a tautomeric rearrangement of quinone (I) into quinodiimine (II) followed by quaternization of nitrogen with the formation of dication (III) and rearrangement of the quinimine ring into an aromatic ring with the formation of a bipolaron (IV) [54,55,56] (Scheme 3):

The unpaired electron on the nitrogen atom is delocalized through a system of conjugated double bonds of the arylamine and hydroquinone groups (IV, V, and VI are part of possible resonance structures). This way, these tautomeric forms are quite stable, and the unpaired electron is not capable of intermolecular recombination. In the presence of an oxidizing agent the one-electron oxidation reaction occurs easily in this monomer with the abstraction of the proton and the regeneration of the quinone structure (VII). During the oxidation, the stabilization of the remaining radical center is disrupted, which makes it capable of recombination. After that, the process continues similarly to the polymerization of aniline and other aromatic amines by recombination of radicals of structures VII and VIII, that is, chain growth proceeds according to the head to tail a coupling type [14]. The mechanism described is designated as the “tautomeric” mechanism.

In such a system, the number of formed radical cations should increase with increasing acid concentration.

With that in mind, it can be assumed that in the case of oxidative polymerization of DADCB, the initial reaction stage is not one-electron oxidation of the nitrogen atom in the aniline group caused by APS, as occurs in the polymerization of aniline, but the formation of radical cations as a result of tautomeric rearrangements, protonation, and oxidation of monomer hydroquinone structures. This explains the lengthening of the induction period under aqueous and alkaline conditions, whereas in the case of aniline it decreases under these conditions. This can also explain the higher reactivity of DADCB, as hydroxy groups with a much lower oxidation potential, and not a nitrogen atom with an electron acceptor substituent, are subjected to oxidation.

**Figure 5:**
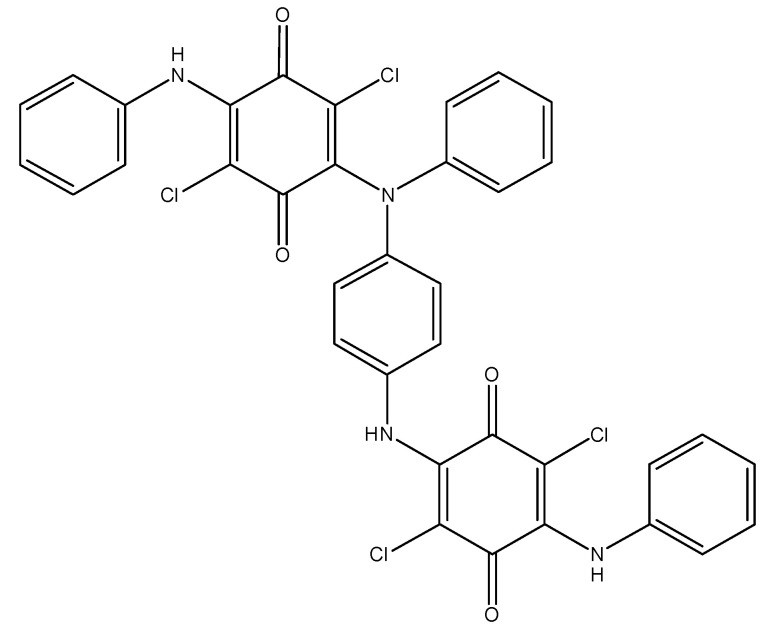
Structure of a DADCB dimer.

As it was already mentioned, only one arylamine group takes part in the oxidation of DADCB. It can be assumed that after the recombination and the formation of a dimer (Figure 5), the part of the dimer molecule that contains the tertiary nitrogen atom is incapable of further tautomeric rearrangements. The other part of the dimer undergoes the same processes as described above and continues the chain growth. As a result, the obtained polymer has the structure of an N-substituted PANI with pendent arylaminochloranil substituents.

### 3.2. Study of the Oxidation of 2-Methoxy-5-anilino-3,6-dichloro-1,4-benzoquinone (MADCB)

To confirm the abovementioned oxidation reaction mechanism, we investigated the oxidative polymerization of a monomer in which the dichloroquinone fragment has only one arylamine substituent. An asymmetrical monomer—MADCB was synthesized via a two-step method through the methoxy derivative of CA followed by alkylation of aniline (Scheme 1II).

**Figure 6:**
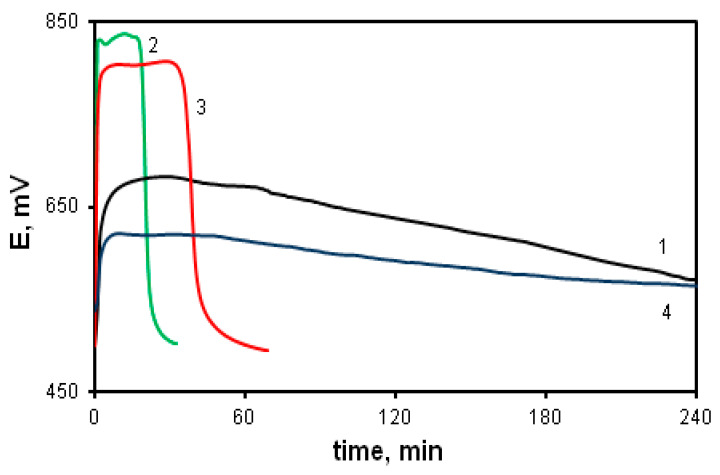
Time dependences of the redox potential during the oxidation reaction of MADCB (1); DADCB (2), and aniline (3) at [HCl] = 0.5 mol/L, as well as MADCB at [HCl] = 1 mol/L (4).

The oxidative polymerization of MADCB was carried out under the standard conditions described above. Figure 6 shows time dependence of the redox potential during the oxidation reaction of MADCB (curve 1). For comparison, the dependences for the polymerization of DADCB and aniline are given (curves 2 and 3).

It can be noticed that MADCB has a much lower oxidation reaction rate than DADCB and aniline. It is difficult to identify the part of the curve related to the induction period typical for aniline polymerization. In the case of MADCB, the methoxydichlorobenzoquinone group demonstrates properties of a strong electron acceptor substituent at the nitrogen atom. Due to this, the reactivity of the arylamine group drops sharply. Besides, bulky substituents at the nitrogen atom reduce the oxidation reaction rate due to the steric factor [40,57,58]. As already mentioned, in some cases, a decrease in the electron density on the nitrogen atom due to electron acceptor substituents leads to a complete inhibition of the polymerization reaction [59].

An increase in the acid concentration from 0.5 M to 1 M not only does not lead to an increase in the reaction rate, as it is observed during the DADCB polymerization, but even slows down the process, as in the case of the oxidation of aniline (curve 4). This indicates that the polymerization of MADCB corresponds to the conventional polymerization process of N-substituted anilines.

Figure S2 shows FTIR spectra of MADCB (1) and poly-MADCB (2). The IR spectrum of MADCB (spectrum 1) clearly demonstrates all spectral signs of the substitution of one of the chlorine atoms in CA with aniline. There is an intense band at 3235 cm^−1^ due to the N-H bond and an intense split band in the area of 1309 cm^−1^ associated with stretching vibrations of the C-N bonds. The bands at 1494–1500 cm^−1^ (associated with C=C stretching vibrations), as well as at 692 and 742 cm^−1^ (associated with C=C-H bending vibrations) refer to the monosubstituted phenyl ring of the aniline substituent [60]. The presence of a methoxy group in the MACB spectrum is characterized by bands at 1195 cm^−1^ and 1290 cm^−1^ (shoulder) corresponding to stretching and bending vibrations of Ph-O-C bonds, as well as at 1445 cm^−1^ associated with C–H vibrations in the –CH_3_ fragment [61,62,63]. The replacement of the electron acceptor substituent -Cl in the chloranil segment with the electron-donor -OCH_3_ leads to a significant redistribution of the electron density in the quinone ring, and, as a consequence, to the delocalization of electrons along the ring and C-Cl and C=O bonds. As a result, a significant shift of the bands occurs: stretching vibrations of C=O bonds from 1672 to 1653 cm^−1^ and C-Cl from 1110 to 1060 cm^−1^ [64,65,66].

**Scheme 4:**
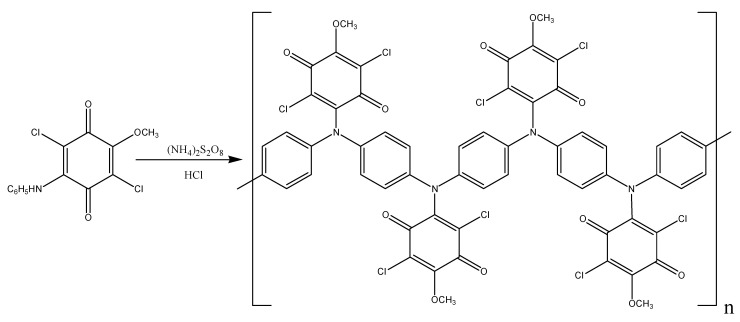
Poly-MADCB synthesis scheme.

The polymer spectrum (spectrum 2) demonstrates intensity growth and band shifts compared to the monomer spectrum in the region of 1150–1250 cm^−1^, typical for the bands of Ph-O-C ether bonds; the ratio of band intensities of C-N bonds in the region of 1309 cm^−1^ changes. This may be due to the fact that different conformations are actualized in the MADCB monomer and poly-MADCB at Ph-N-CA and CA-O-Me nodes. This is also indicated by the fact that the bands of C=O and C-Cl bonds in pendent quinoid substituents of the polymer are split and there appear bands similar to such bands in the CA spectrum, whereas in the monomer spectrum these bands are not split and are shifted towards long wavelengths [65,66]. It is easy to imagine that an electron-donor sterically bulky substituent appearing in the polymer structure in CA units with –I (inductive) and +M (mesomeric) effects can lead to rotations in the Ph-N-CA and CA-O-Me nodes. In this case, there is a redistribution of the electron density in the quinone ring with a higher localization of electrons on the C=O and C-Cl bonds. Thus, it can be assumed that the quinone ring in the MADCB monomer has a delocalized electronic structure resembling a benzoic one, whereas the quinone rings in the polymer, due to conformational rearrangements in the Ph-N-CA and CA-O-Me nodes, has an electronic structure that gets nearer to CA. In the spectrum of poly-MADCB (spectrum 2), new bands at 823 cm^−1^ and 1027 cm^−1^ appear, typical for δ_C(Ph)=CH_ 1,4-substituted aromatic rings [60,67], which is a clear indication of the polymer structure shown in Scheme 4. 

At the same time, a broad intense band of N-H bonds at 3325 cm^−1^ and bands of monosubstituted aromatic rings at 692 and 742 cm^−1^ are retained. This leads to a conclusion that the polymerization degree of the polymer is low and the polymer retains a sufficient amount of terminal aniline units [60]. Thus, it can be assumed that oligomerization of MADCB has the “head-to-tail” coupling type, as in the case of aniline. The polymer molecular weight is M_w_ = 2.1 × 10^3^ g/mol.

### 3.3. The Effect of Substituent Types in Arylamine Groups of the Quinone Ring on the Oxidation Reaction Rate and the Structure of the Resulting Polymers

The oxidative polymerization of monomers with electron-donor and electron-acceptor substituents in the meta-position of the benzene ring was studied in comparison with the polymerization of DADCB and aniline.

#### 3.3.1. Oxidative Polymerization of 2,5-Diphenylenediamino-3,6-dichloro-1,4-benzoquinone (DPDCB)

**Figure 7:**
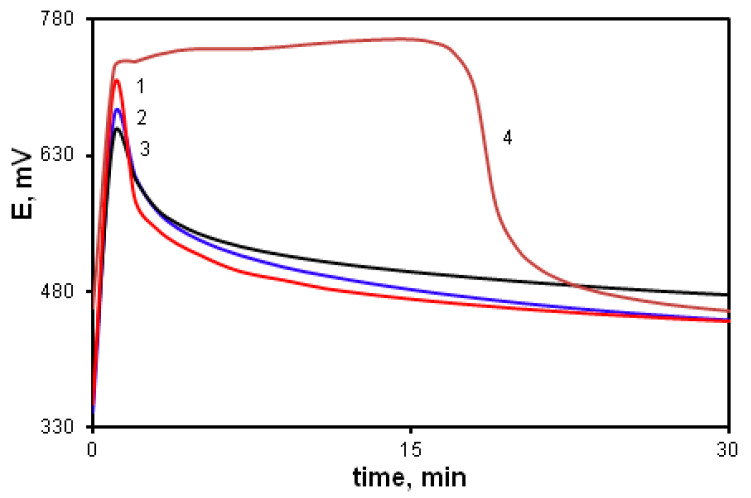
Time dependence of the redox potential during the oxidation reaction: [DPDCB] = 0.05 mol/L (1), 0.03 mol/L (2), 0.01 mol/L (3); [DADCB] = 0.03 mol/L (4).

Figure 7 shows time dependences of the redox potential during the polymerization of DPDCB at various monomer concentrations (curves 1–3). For comparison, the DADCB polymerization curve is also shown (curve 4). It can be seen that at the same monomer concentration (curves 2 and 4), the oxidation reaction rate of DPDCB is much higher than that of DADCB. At the same time, there is no induction period of the reaction. The kinetic curves are not S-shaped as would be typical for the oxidative polymerization of aniline.

The explanation for this character of polymerization can lie in the fact that a DPDCB molecule contains additional amino groups, which significantly increases the electron density in the para-position of the aniline ring. This leads to an increase in its nucleophilicity and in monomer reactivity during the chain growth of by electrophilic substitution type. As a result, the induction period is completely absent (Figure 7, curves 1, 2, 3), unlike the curves of the polymerization of DADCB and aniline.

However, the oxidative polymerization of DPDCB differs visually not only from the polymerization of aniline, but also from the polymerization of DADCB. When the reaction is complete, the solution does not acquire the dark green color typical for the emeraldine form of PANI. During the synthesis, the reaction mixture darkens quickly and a finely dispersed dark gray powder precipitates. Besides, the resulting product does not have “blue/green” color changes typical for PANI structures during acid-base treatment. This indicates that the oxidative polymerization of DPDCB proceeds with the formation of polymer structures different from both PANI and DADCB polymers. This is confirmed by FTIR and XPS data. 

Figure S3 shows FTIR spectra of DPDCB and its oxidation product. The monomer spectrum fully corresponds to the structure of disubstituted chloranil. The presence of chlorine in the chloranil nucleus substituted with two phenylenediamine substituents is well confirmed by intense bands at 1112 and 873 cm^−1^ corresponding to C-Cl bonds in the quinoid ring [51,68,69]. The bands corresponding to C=O bonds in the CA spectrum at 1680–1677 cm^−1^ in the monomer spectrum are shifted to long wavelengths and, together with the bands corresponding to stretching vibrations of aromatic substituents and to bending vibrations in the amino group, give an intense split band at 1624 cm^−1^ [51,64,70]. Phenylenediamine substituents cause bands corresponding to amino groups at 3216, 3341 cm^−1^ (νNH), 1311 cm^−1^ (νC-N), 1574 and 1496 cm^−1^ (νC=C(Ph) in the monomer spectrum, as well as two bands at 685 and 803 cm^−1^ that are well comparable with out-of-plane bending vibrations of δCCH in the 1,3-substituted aromatic ring [48,50,71,72]. Broad bands in the region of 2000, 2810, and 2585 cm^−1^ characterize the ammonium cation and indicate the salt form of the obtained monomer. Since HCl is evolved during the synthesis of the monomer, it partially protonates the primary amino group of the phenylenediamine ring.

The polymers were studied in a dedoped form, that is why the IR spectrum of the polymer shows no spectral signs of ammonium cations. The bands corresponding to stretching vibrations of N-H bonds in amino groups (3216, 3341 cm^−1^), as well as the band at 1311 cm^−1^ corresponding to stretching vibrations of C-N- bonds in the polymer spectrum appear at the same wavenumbers as in the monomer spectrum. Only the relative intensity of these bands changes, and the band at 1311 cm^−1^ appears as a shoulder of the intense band at 1269 cm^−1^. This indicates that some part of phenylenediamine groups remains unchanged after oxidative polymerization. The band at 1624 cm^−1^ in the polymer spectrum disappears completely. Instead, a very intense split band appears at 1606 cm^−1^, which can be an overlap of bands corresponding to C=C(Ph) stretching vibrations, H-N-H bending vibrations of the amino group, and a band associated with stretching vibrations in conjugated C=O, -C=N- bonds which usually has a very high intensity [73]. A highly intense band at 1269 cm^−1^ corresponding to (Ph)C-O-C(Ph) asymmetric stretching vibrations [74,75] can be regarded as a spectral sign of the presence of phenoxazine structures in the polymer structure. The absence of intense bands at 1112 and 873 cm^−1^ corresponding to C-Cl bonds in the polymer spectrum is a direct indication of a sharp decrease in the amount of chlorine in the polymer structure. The polymer spectrum contains weak bands at 685 and 803 cm^−1^ corresponding to 1,3-substituted aromatic rings. Their low intensity indicates a significant decrease in the proportion of 1,3-substituted phenyl rings in the polymer as compared to the monomer. The new broad intense band at 830 cm^−1^ in the polymer spectrum indicates the presence of a large number of 1,2,4,5-substituted aromatic rings in the polymer, resulting from cyclization reactions [37,76,77].

A detailed analysis of the X-ray photoelectron spectra of DPDCB and poly-DPDCB confirms the FTIR spectroscopy data.

As seen from Table 1, the chlorine content is halved. This indicates that half of the chlorine is removed being substituted by an amino group with the possible formation of phenazine rings, which is confirmed by a detailed analysis of the approximation parameters of O1s, Cl2p, N1s, C1s high-resolution spectra (Table S2 and Figures S4 and S5).

The O1s spectrum of the monomer contains two peaks at 531.6 eV and 533.3 eV with the intensity ratio of 85:15. The first peak can be attributed to the oxygen of the C=O bond and the second to the C-OH bond [78]. This indicates that the monomer is predominantly in the quinone form. For the polymer, the ratio of these two peaks at 531.5 and 533.2 eV is 50:50. Since tautomeric rearrangements are impossible in polymers, it should be assumed that half of the oxygen in the DPDCB polymer remains in the quinone form, while the rest creates an additional bond with the second carbon atom, forming the C-O-C group [79].

The N1s nitrogen spectra of the monomer contain the main peak associated with nitrogen bound to ring atoms (399.9 eV). A low-intensity peak (401.65 eV) indicates the presence of a small amount (12%) of N^+^‒H quaternized nitrogen. In the DPDCB polymer, this peak (401.7 eV) decreases to the background level. The presence of charged nitrogen in the monomer is also confirmed by IR spectra. It remains in the monomer as a result of the synthesis that proceeds with the release of HCl. In the N1s spectrum of the DPDCB polymer, a shoulder appears in the region of 398.6 eV (23%), which can be attributed to the nitrogen of the phenazine ring.

Chlorine spectra are spectra of 2p3/2-2p1/2 doublets with a splitting of 1.6 eV. The main doublet at 200.86 eV corresponds to C-Cl bonds. The second doublet at 197.4 eV corresponds to NH^+^Cl^−^ and its intensity in the polymer decreases to 4%.

When moving from monomer to polymer, C1s carbon spectra show a decrease from 43 to 32% in the intensity of the 284.7 eV peak, which corresponds to aromatic carbons not associated with nitrogen, oxygen or chlorine. This decrease is significantly greater than the aniline polymerization scheme could suggest. It should be assumed that some carbon atoms are replaced as a result of the heterocycle formation, which is confirmed by the increased intensity of the peak at 286.5–286.4 associated with C=N and C-O-C. An increase in the total intensity of the peaks at 285.8 (C-N) and 286.4 eV from 43 to 60%, and a two-fold decrease in the amount of C=O (288.9 eV) correspond to the growth of polycyclic structures.

**Figure 8:**
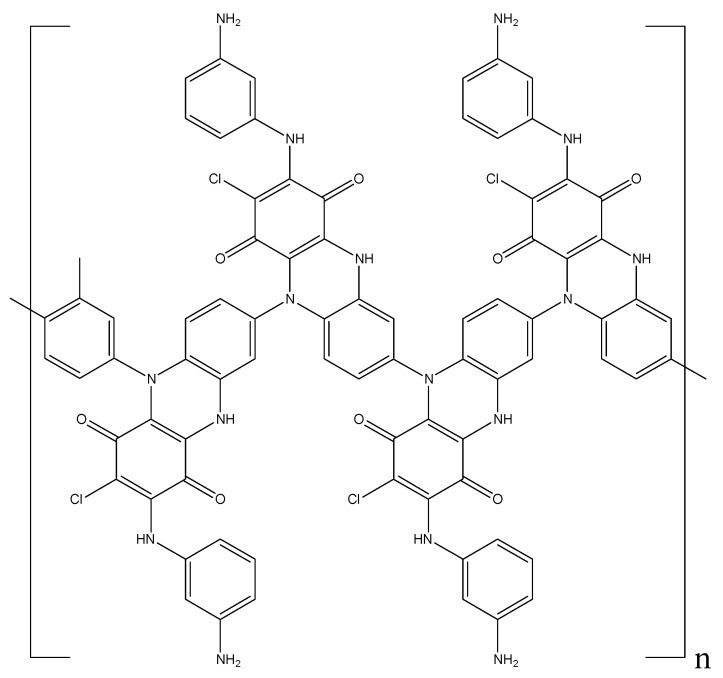
Intermediate polymer structure with hydrophenazine rings.

During the polymerization process, the pendent amino group located in the ring on the polyaniline chain is in close proximity to the chlorine atoms of the quinone ring. This facilitates the nucleophilic substitution reaction with the formation of substituted hydrophenazine rings (Figure 8).

**Figure 9:**
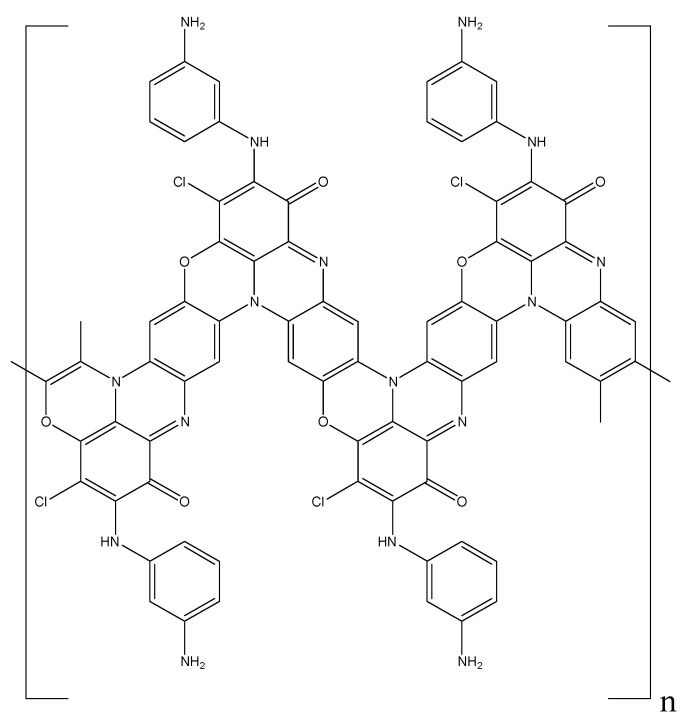
Structure of poly-DPDCB.

However, due to their instability, they are immediately oxidized by APS. At the same time, a rearrangement of the system of conjugated double bonds not only of phenazine, but also quinone rings takes place. The activation of oxygen of the quinone ring due to an increase in its electrophilicity and an increase in the nucleophilicity of para-position of the benzene ring located on the other side of the phenazine ring leads to the formation of a phenoxazine ring with an orthoquinoimine structure (Figure 9). As a result, the forming conjugated polymer has a ladder structure where, along with the PANI chain common for the polymerization of aromatic amines, phenazine and phenoxazine heterocyclic fragments are also present. At the same time, the content of chlorine atoms is two times smaller compared to the monomer, and the ratio of the carbonyl oxygen of quinone and the oxygen of the ether group in the phenoxazine ring is 50:50.

The polycyclic nature of the resulting DPDCB polymer prevents its PANI-type doping and leads to the absence of typical color transitions in the processes of acid-base treatment. The absence of a green or blue-purple color of the reaction mixture during synthesis indicates that the cyclization reaction proceeds simultaneously during the growth of polymer chains, and by the end of the reaction the polymer already has a polycyclic structure. Poly-DPDCB is not completely soluble. The molecular weight of its soluble fraction in N-MP is M_w_ = 2.14 × 10^4^ g/mol.

**Figure 10:**
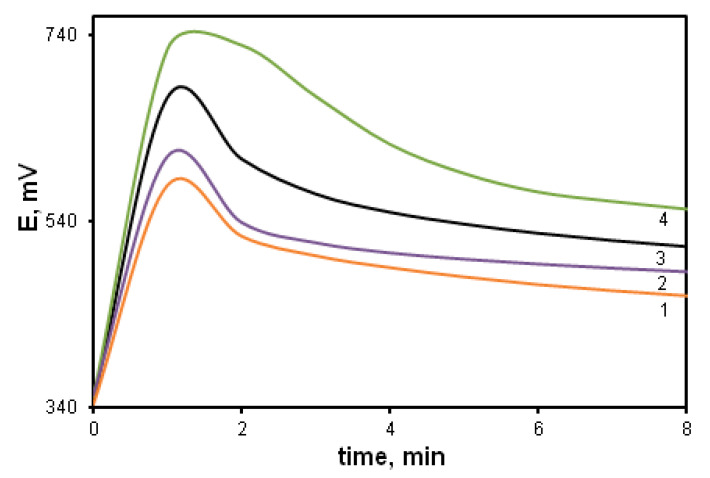
Time dependence of the redox potential during the DPDCB oxidation at different ratios of [Ox]/[DPDCB]: 0.5 (1), 0.75 (2), 1.25 (3), 3.75 (4).

The effect of the oxidant/monomer ratio during the oxidative polymerization of DPDCB (Figure 10) was studied. With a decrease in the oxidant/monomer ratio below the stoichiometric ratio (1.25) down to values of 0.75 (curve 2) and 0.5 (curve 1), the reaction rate decreases. However, with an increase in the oxidant/monomer ratio above the stoichiometric ratio, no lengthening of the induction period typical for the polymerization of aniline and DADCB is observed. Only when there is a significant increase in the ratio up to 3.75 (curve 4), a slight decrease in the reaction rate is observed and the induction period is retained. This is due to the fact that part of the oxidant is spent on cyclization processes.

**Figure 11:**
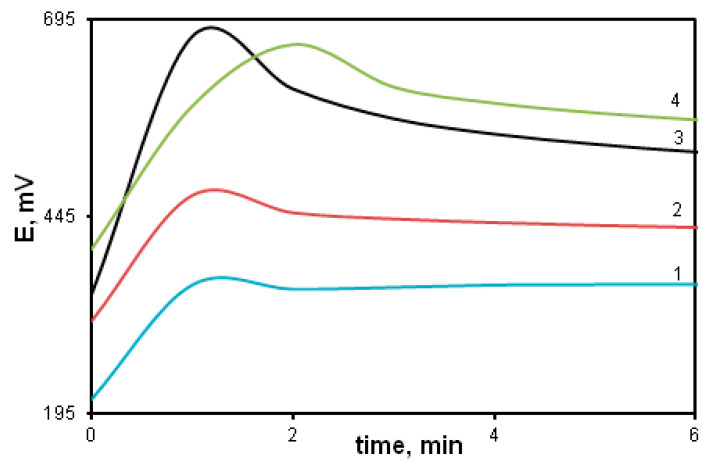
Time dependence of the redox potential during the DPDCB oxidation in different media: water (1), [HCl] = 0.1 mol/L (2), 0.5 mol/L (3); 1 mol/L (4).

The effect of the reaction solution pH on the reaction rate is shown in Figure 11. Comparison of the potentiometric curves indicates that the reaction rate grows with the decrease in pH. The intensity of the peak also increases (curves 1, 2, 3). This indicates that the concentration of radical cation centers is directly related to the acid concentration in the reaction solution in accordance with the “tautomeric” reaction mechanism. However, when acid concentration is above 0.5 M (curve 4), the induction period begins to grow and the reaction rate falls significantly. This is due to the fact that an excess of acid protonates the primary amino groups of the phenylenediamine rings, converting them into electron-acceptor substituents that reduce electron density in the para-position of the aniline ring. Its nucleophilicity goes down and its reactivity decreases.

#### 3.3.2. Oxidative Polymerization of 2-(Aniline-3-sulfo)-5-anilino-3,6-dichloro-1,4-benzoquinone (ASADCB)

**Figure 12:**
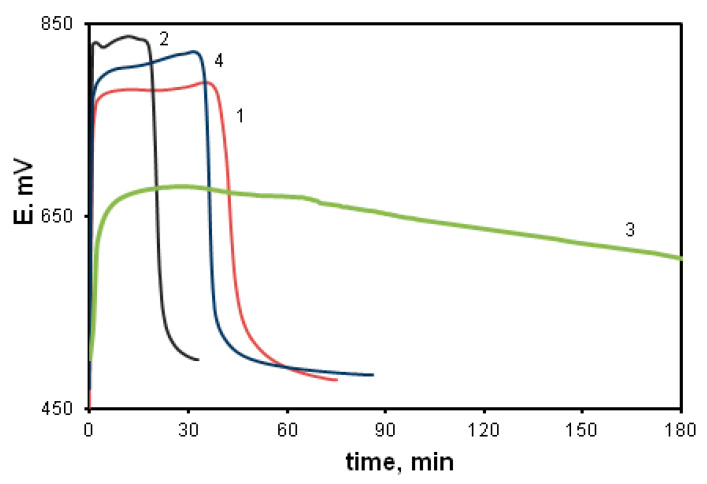
Time dependence of the redox potential during the oxidation of ASADCB (1), DADCB (2) and MADCB (3) at [HCl] = 0.5 mol/L, and ASADCB (4) at [HCl] = 1 mol/L.

The effect of the acceptor substituent in the arylamino group was studied using the oxidative polymerization of ASADCB as an example. It is known that the presence of a sulfo group turns substituted aniline inactive in the oxidative polymerization reaction [59]. However, we carried out the polymerization of ASADCB with a sulfo group in the aniline ring. Figure 12 shows kinetic curves corresponding to the oxidative polymerization of ASADCB and, for comparison, of DADCB and MADCB.

It can be seen that the induction period is longer, and the reaction rate is slightly lower than that of the monomer without substituents (DADCB). Yet, as in the case of DADCB polymerization, the increase in the acid concentration leads to the shortening of the induction period and the decrease in the oxidation rate (curve 4). However, the reaction rate is much higher than during the polymerization of the asymmetric MADCB monomer described above, which polymerizes as a substituted aniline. Despite the fact that the substituted arylamine group with a sulfo substituent does not participate in the polyrecombination reaction and the polymer chain growth is rather rapid, it can be assumed that the polymerization of ASADCB proceeds similarly to the oxidation of DADCB. The lengthening of the induction period and a slight decrease in the oxidation rate compared to those of DADCB can be explained by the influence of symmetry disruption on stability of tautomeric forms and, consequently, on the concentration of active radical cation centers. This can also explain the decrease in molecular weight in comparison with DADCB. The molecular weight of poly-ASADCB does not exceed M_w_ = 1.7 × 10^4^ g/mol.

Figure S6 shows ASADCB (1) and poly-ASADCB polymer (2) FTIR spectra.

The ASADCB spectrum demonstrates spectral signs of aniline addition: a strong increase in band intensity in the region of 1500 cm^−1^ corresponding to skeletal vibrations of the aromatic ring and at 694 cm^−1^ corresponding to out-of-plane bending vibrations of the monosubstituted aromatic ring, which the new band at 720 cm^−1^ also refers to. There is a sharp drop in the intensity of bands at 617, 1192, 1045 cm^−1^ that characterize the sulfo group. Split bands in the range of 1567–1485 cm^−1^ refer to benzoic and quinone rings. The band corresponding to the N-C bond in the region of 1310–1328 cm^−1^ splits and grows in intensity in the ASADCB spectrum. In the ASADCB spectrum, the isolated N-H bond causes a band at 3225 cm^−1^, and there are also weak signs of association between N-H and O-H bonds of the sulfo group (weakly intense broad bands in the region of 3100–2600 cm^−1^). In the asymmetric ASADCB monomer, the sterically looser N-H bond in the aniline ring is partially associated with the sulfo group of another molecule. A new rather intense band at 829 cm^−1^ in the polymer spectrum is a direct indication that p-substituted aromatic rings that were absent in the ASADCB monomer sample appear in the poly-ASADCB sample [60]. There is also a slight growth of the band at 1495 cm^−1^ in the polymer spectrum, which also indicates an increase in the proportion of substituted phenyl rings.

**Scheme 5:**
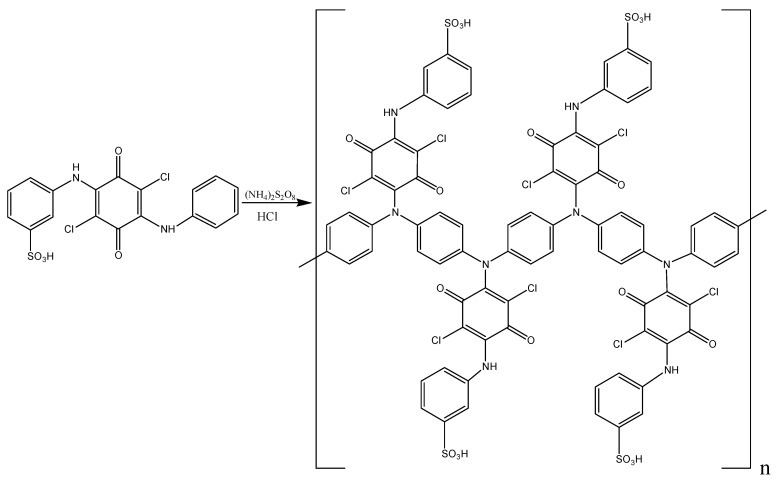
Poly-ASADCB synthesis scheme.

The structure of the obtained poly-ASADCB corresponds to that of PANI, where the linkage is of the head-to-tail type. At the same time, the polymer demonstrates no signs that the sulfo group takes part in the act of addition, since the polymer spectrum does not contain new bands in the range of 810–750 and 710–690 cm^−1^ common for 1,3,4-substituted aromatic rings. Additionally, the bands at 623, 1040 and 1200 cm^−1^, typical for the sulfo group [80], are not changed in the spectrum of the monomer and polymer. This means it remains inactive and does not prevent tautomeric transformations. The spectra of the monomer and polymer in the Figure S6 correspond to the structures in Scheme 5.

We failed to carry out the polymerization of 2,5-di(aniline-3-sulfo)-3,6-dichloro-1,4-benzoquinone (ASDCB)—the monomer with sulfo groups in each arylamine substituent. The oxidative polymerization of ASDCB was carried out under the same conditions, standard for the abovementioned monomers. However, it was not possible to show—neither visually nor potentiometrically—that the polymerization process is in progress.

Thus, the process of the oxidative polymerization of diarylaminodichlorobenzoquinones depends on whether the benzene ring has an electron donor or electron acceptor substituent. Probably, the effect caused by the substituent refers to the para-position of the benzene ring responsible for polyaddition rather than to the active center of polymerization—the nitrogen atom. This assumption is confirmed by the fact that the DPDCB polymerization rate decreases when all nitrogen atoms are in the quaternized form. Then the primary amino group in the aromatic ring ceases to have the electron-donor effect, reducing the nucleophilicity in the para-position of the benzene ring and decreasing its reactivity. The electron acceptor sulfo group in the arylamine ring of ASADCB reduces the reactivity of the para-position of the benzene ring. Oxidative polymerization is completely blocked at any concentration of the monomer, oxidant, acid, when the monomer structure contains sulfo groups in each arylamine ring, as it is in ASDCB.

Thus, if a monomer of the diarylaminodichlorobenzoquinone type contains two arylamine groups in the quinone ring and at least one of them does not block the polymer chain growth, the reaction will proceed according to the “tautomeric mechanism” with a higher oxidation rate compared to the oxidation of aniline.

### 3.4. Some Properties of the Obtained Polydiarylaminodichlorobenzoquinones

**Figure 13:**
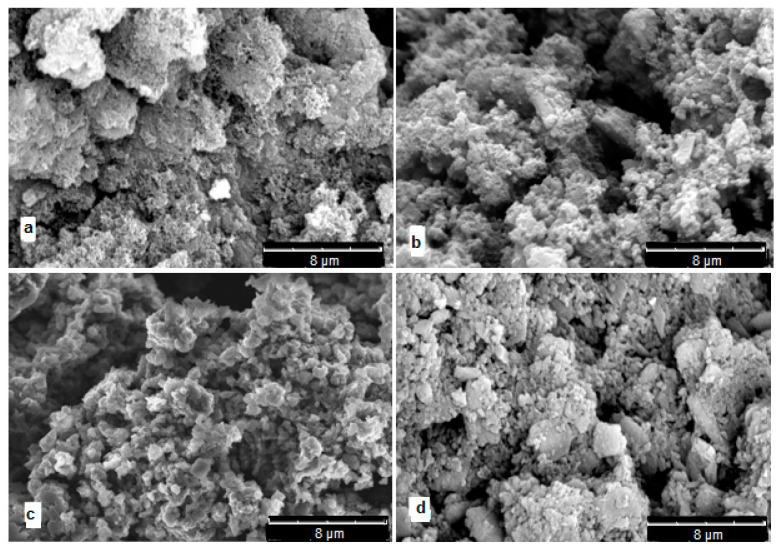
SEM micrographs: (**a**)—PANI, (**b**)—poly-DADCB, (**c**)—poly-DPDCB, (**d**)—poly-ASADCB.

All synthesized polymers are dark gray-green powders. Figure 13 shows SEM micrographs of polymers. It can be seen that all samples are loose globular morphology not very different from PANI. It can only be noted that globules in polydiarylaminodichlorobenzoquinones have larger size and their structure seems to be denser.

According to X-ray structural analysis data, all the obtained polymers are amorphous.

The electrical conductivity σ_dc_ and σ_ac_ of the synthesized polymers was studied. The frequency dependence on the conductivity σ_ac_ is described by the equation:

σ_σ__c_ = σ_dc +_ Aω^n^, (1)

where ω = 2πf is the angular frequency,

σ_dc_—the frequency independent part of conductivity,
n—the exponential parameter (0 ≤ n ≤ 1),
A—the thermally activated quantity.

A and n depend on the temperature and the volume fraction of the conducting component.

**Figure 14:**
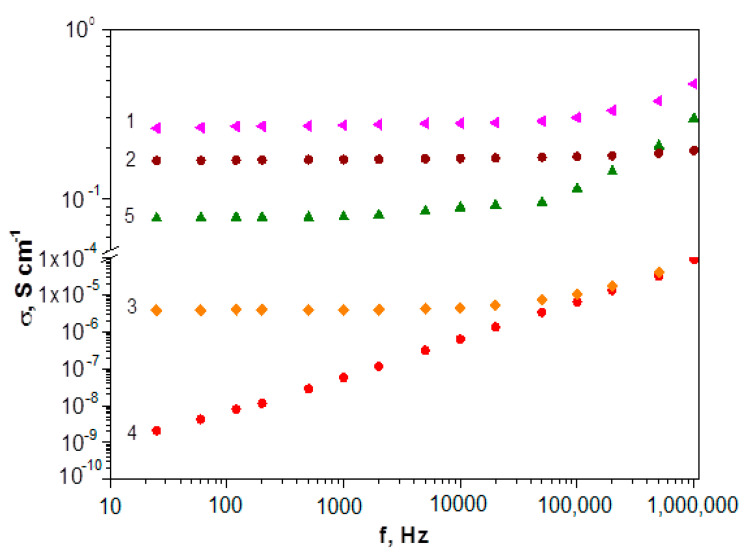
Current frequency dependence of conductivity: poly-DADCB (1), PANI (2), poly-DPDCB (3), poly-MADCB (4), poly-ASADCB (5).

Figure 14 shows frequency dependence of conductivity. It can be seen that frequency dependence σ_ac_ shows the growth of electrical conductivity with the increase in the current frequency for all polymers. It indicates that the tunneling effect plays a minimal role [81].

Table 2 shows the values of σ*_dc_*, as well as values σ*_ac_* and *n*.

As can be seen from the table, the exponential parameter *n* is in the range of 0 < *n* < 1 for all studied polymers, which is typical for disperse systems with a hopping charge carrier transport mechanism [81,82,83]. The σ_dc_ value plays a significant role at low frequencies. Its growth leads to a high-frequency shift of the onset of the increase in the electrical conductivity of polymers. It should be noted that polydiaryldichlorobenzoquinones with the structure of N-substituted PANI (poly-DADCB, poly-ASADCB) are characterized by higher σ_ac_ values than the poly-DPDCB, which has a ladder structure with phenazine and phenoxazine rings. This can be caused both by a low degree of conjugation due to the presence of defect structures and by a low probability of the formation of a doped polaron structure. At the same time, if the electrical conductivity of poly-ASADCB is close to the conductivity of PANI obtained under the same conditions, then the electrical conductivity of poly-DADCB exceeds the value of the electrical conductivity of PANI. As it was shown in the introduction, previously synthesized N- and C-substituted PANI are characterized by very low electrical conductivity values. It can be assumed that the formation mechanism of active polymerization centers described above determines the formation of more extended regions of continuous conjugation that are responsible for the polymer electrical conductivity and the degree of their order. This is confirmed by the very low electrical conductivity values of poly-MADCB, which is N-substituted PANI with electron-acceptor substituents in the pendent chain.

## 4. Conclusions

The oxidative polymerization of a new class of monomers—diarylaminodichlorobenzoquinones—was studied for the first time. Monomers were obtained via alkylation of aniline and its derivatives with chloranil. Symmetric DADCB, DPDCB and DASDCB monomers were obtained in a one-step synthesis via alkylation of aniline, meta-phenylenediamine and metanilic acid with chloranil. A two-stage sequential alkylation method was used to synthesize asymmetric monomers in which the substituents on the chloranil ring were different—MADCB and ASADCB.

It was found that the oxidative polymerization of monomers of the diarylaminodichlorobenzoquinone type does not follow the main features of polymerization of substituted anilines. First of all, they have a higher oxidation rate compared to aniline and its derivatives. Despite the fact that the chloranil substituent is electron acceptor, its presence did not inhibit the oxidation reaction, but rather accelerated it significantly at the initial stage of the induction period. It shortened considerably or disappeared altogether, as in the case of DPDCB. Besides, a specific feature of the oxidative polymerization of diarylaminodichlorobenzoquinones was the participation of only one arylamine group in the reaction, even though most of the studied monomers had two reaction centers. The optimal oxidant/monomer ratio is stoichiometric for one arylamine group, despite the bifunctionality of the monomer. It is shown that the oxidation rate increases with an increase in the acid concentration in the reaction solution.

The analysis of the obtained experimental results allowed us to propose a formation mechanism for active polymerization centers diarylaminodichlorobenzoquinones. Its essence is the ability of these structures to undergo tautomeric rearrangements, sequentially leading from diarylaminodichlorobenzoquinone to quinodiimine structures that, in the process of further protonation, lead to the rearrangement of the quinodiimine ring into an aromatic ring with the formation of radical cations on nitrogen atoms. Further one electron oxidation leads to the reduction in the quinone ring and activation of the remaining radical cation center. This mechanism explains the induction period reduction and an increase in the oxidation rate with the decrease in the reaction solution pH. The chain growth occurs similarly to aniline via polyrecombination by “head to tail” type adding.

It was found that for the reaction to proceed according to the “tautomeric” mechanism, the monomer structure should contain two arylamine groups at 2,5-positions of the quinone ring, capable of forming a symmetric quinodiimine structure. Otherwise, as shown by the polymerization of MADCB, the reaction proceeds according to the mechanism common for anilines with electron acceptor substituents, characterized by a long induction period and a low oxidation rate.

It was shown that the nature of the substituent in the aniline ring (electron donor or electron acceptor) determines the polymer chain growth rate. An additional amino group in the meta-position of the arylamine ring (DPDCB) accelerates polymerization, and a sulfo group (DASDCB) inhibits the process. The type of the substituent determines the nucleophilicity degree in the para-position of the benzene ring and, consequently, its reactivity in the chain growth process that occurs according to the electrophilic substitution type. If the monomer contains two different arylamine groups (ASADCB), the polymer chain growth proceeds with the participation of only the group that has no electron-acceptor substituent. In this case, there is a decrease in the polymerization rate probably associated with the disruption of the electronic structure symmetry of the quinoimine ring and, consequently, the stability of its tautomeric forms and the concentration of radical cation centers.

DPDCB was used as an example to show that its amino groups, chlorine atoms and oxygen atoms of the quinone ring take part in further reactions during the polymerization process. For example, the pendent amino group located in the ring on the polyaniline chain, while interacting with chlorine atoms of the quinone ring, leads to the formation of phenazine rings. Additionally, the oxygen of the quinone ring, reacting with the activated benzene ring, leads to the formation of the phenoxazine ring of the orthoquinoimine structure. As a result, a ladder polymer is formed during the oxidative polymerization of DPDCB.

All obtained polymers are amorphous and have a globular morphology. Frequency dependences of electrical conductivity were studied for all synthesized polymers. It was shown that the hopping charge carrier transfer mechanism is present. The electrical conductivity of the polymer based on MADCB corresponds to the values for PANI with bulky electron-acceptor substituents. It is quite low. Polydiaryldichlorobenzoquinones with the structure of N-substituted PANI (poly-DADCB, poly-DASADCB) are characterized by higher electrical conductivity values than the poly-DPDCB, which has a ladder structure with phenazine and phenoxazine rings.

Thus, this article presents the first results of the investigation of the oxidative polymerization of diarylaminodichlorobenzoquinones with the formation of new conjugated polymers. Further development of research in this area will be directed to the design of new polymers of this class and composites based on them, investigation of their properties and determination of their potential for practical application, as well as to the deeper understanding of the fundamental structure-property relationships.

## Figures and Tables

**Table 1:** Elemental analysis data (XPS). Concentration, at. %.

Sample	C	N	O	Cl	O/C	N/C	Cl/C
DPDCB	71.2	15.3	7.1	6.4	0.1	0.21	0.09
poly-DPDCB	72.0	16.7	8.2	3.1	0.11	0.23	0.04

**Table 2:** Electrical properties of polymers.

Sample	σ_dc_, S/cm	σ_ac_ (25 Hz)	σ_ac_ (1 MHz)	n
PANI	1.7 × 10^−1^	1.8 × 10^−1^	3 × 10^−1^	0.39
poly-DADCB	2.6 × 10^−1^	2.6 × 10^−1^	4.8 × 10^−1^	0.79
poly-MADCB	1.4 × 10^−9^	1.8 × 10^−9^	9.2 × 10^−5^	0.99
poly-DPDCB	3.6 × 10^−6^	5.4 × 10^−^^7^	1.1 × 10^−4^	0.99
poly-ASADCB	7.7 × 10^−2^	7.7 × 10^−^^2^	2.9 × 10^−1^	0.76

## Data Availability

The data presented in this study are available on request from the corresponding author.

## Supplementary Materials

The following are available online at https://www.mdpi.com/article/10.3390/polym13213657/s1, Table S1: Properties of polymers, Figure S1: FTIR spectra of DADCB (1) and poly-DADCB (2) in a dedoped form, Figure S2: FTIR spectra of MADCB (1) and poly-MADCB (2) in a dedoped form, Figure S3: FTIR spectra of DPDCB (1) and poly-DPDCB (2) in a dedoped form, Figure S4: C1s (a) N1s (b), O1s (c) Cls (d) DPDCB spectra (XPS), Figure S5: C1s (a) N1s (b), O1s (c) Cl1s (d) poly-DPDCB spectra (XPS), Table S2: Approximation parameters of high-resolution O1s, Cl2p, N1s, C1s spectra (XPS), Figure S6: Comparison of ASADCB (1) and poly-ASADCB (2) FTIR spectra.
