# Supplementary material for: Peculiarities of Oxidative Polymerization of Diarylaminodichlorobenzoquinones
Source: Polymers (Basel). 2021 Oct 23;13(21):3657. doi: 10.3390/polym13213657 (PMC8587632; doi:10.3390/polym13213657)
Supplement: Supplementary file 1 [file polymers-13-03657-s001.zip › polymers-1414389-supplementary.pdf]

polymers

MDPI

Article

Peculiarities of oxidative polymerization of diarylaminodi-chlorobenzoquinones

Andrey V. Orlov, Svetlana G. Kiseleva, Galina P. Karpacheva* and Dmitriy G. Muratov

A.V. Topchiev Institute of Petrochemical Synthesis, Russian Academy of Sciences, Leninsky pr., 29, 119991 Moscow, Russia; avorlov@ips.ac.ru, skisel@ips.ac.ru, muratov@ips.ac.ru

* Correspondence: gpk@ips.ac.ru

Table S1. Properties of polymers.

Sample	Polymerization conditions	M_w, g/mol	PDI	Yeld, %
**poly-DADCB**	standard*	2.72 × 10^4	1.2	96
**poly-MADCB**	standard	2.1 × 10^3	1.4	87
**poly-DPDCB**	standard	2.14 × 10^4	1.8	92
**poly-ASADCB**	standard	1.7 × 10^4	1.5	89

* - Standard polymerization conditions had the following parameters: [monomer] = 0.03 mol/L; [APS]/[monomer] = 1.25; [HCl] = 0.5 mol/L; T = 20 °C

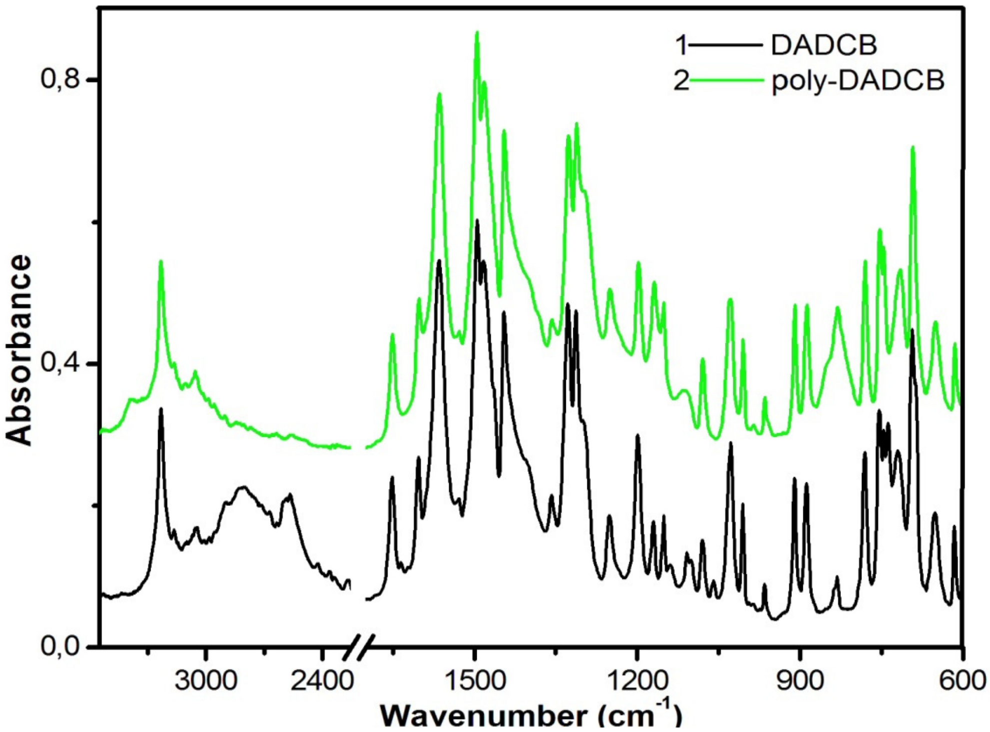

Figure S1. FTIR spectra of DADCB (1) and poly-DADCB (2) in a dedoped form.

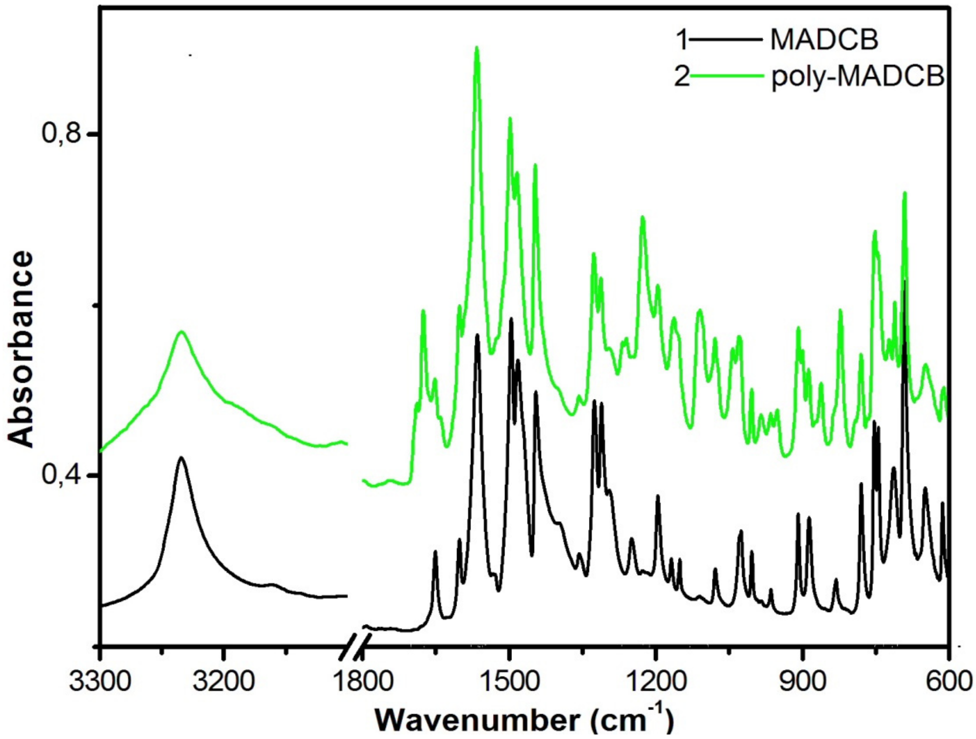

Figure S2. FTIR spectra of MADCB (1) and poly-MADCB (2) in a dedoped form.

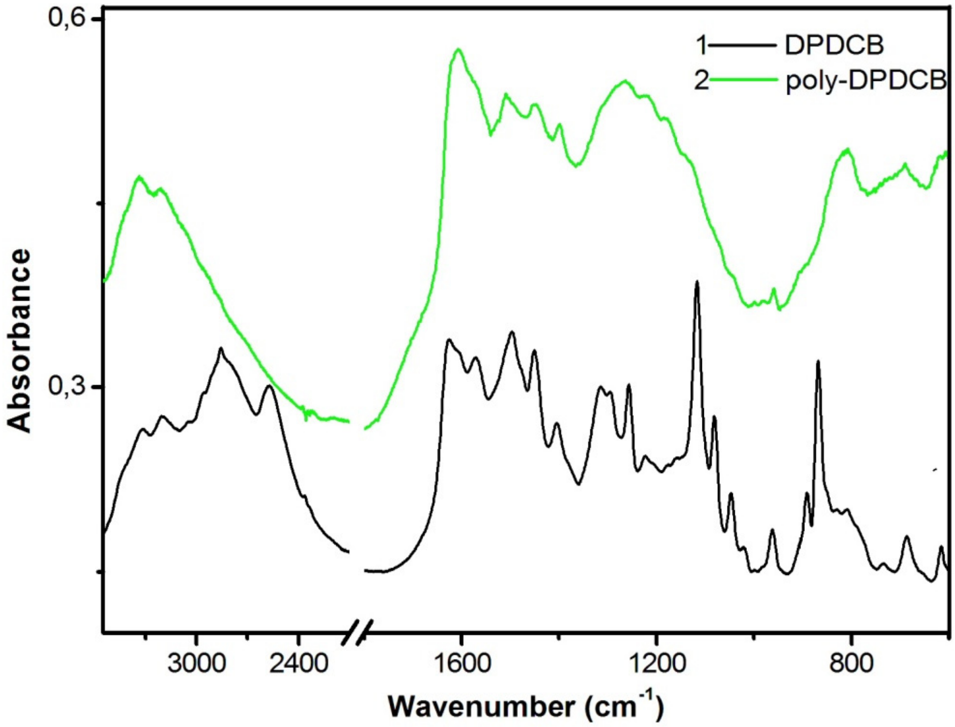

Figure S3. FTIR spectra of DPDCB (1) and poly-DPDCB (2) in a dedoped form.

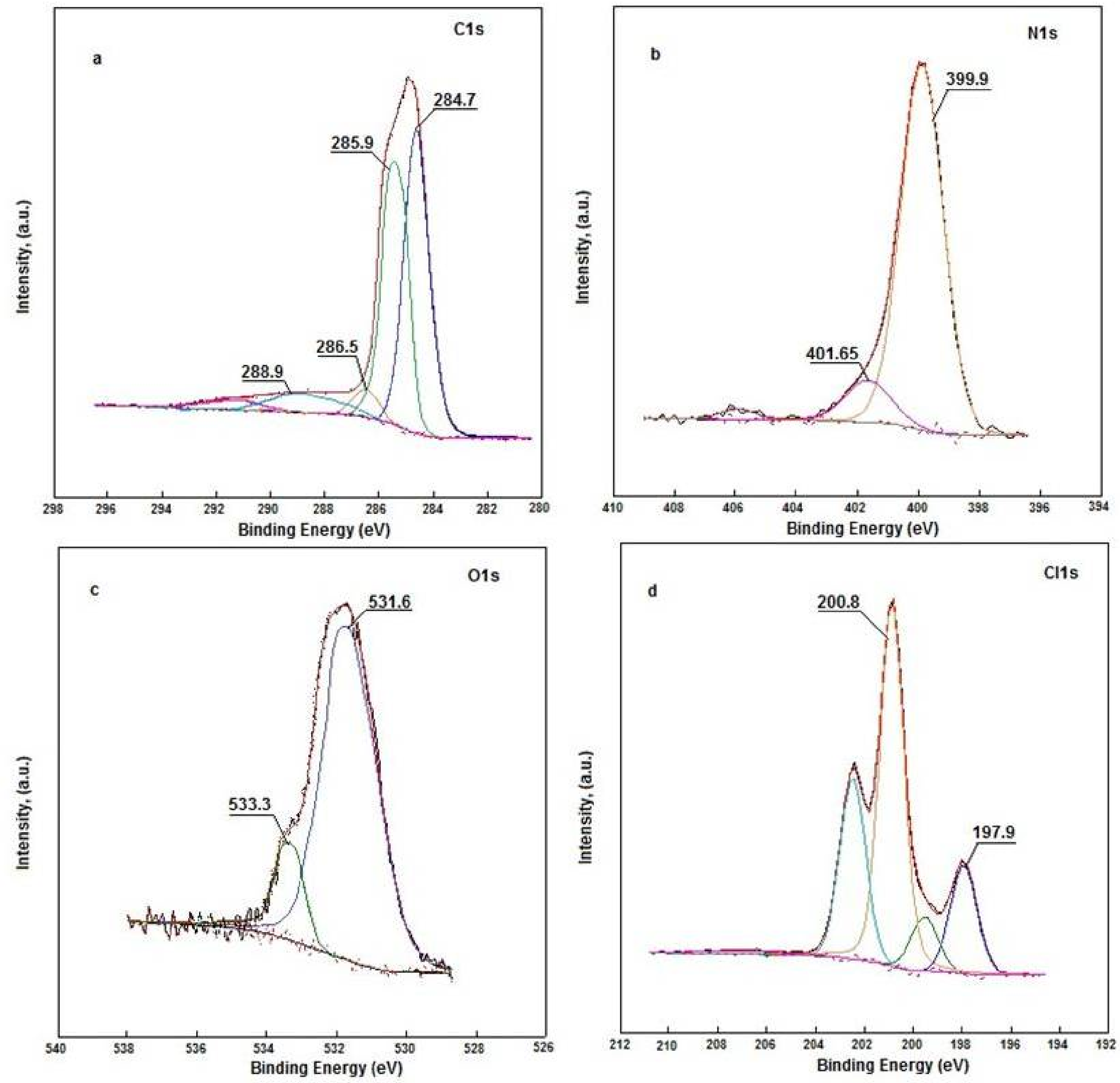

Figure S4. C1s (a) N1s (b), O1s (c) Cls (d) DPDCB spectra (XPS).

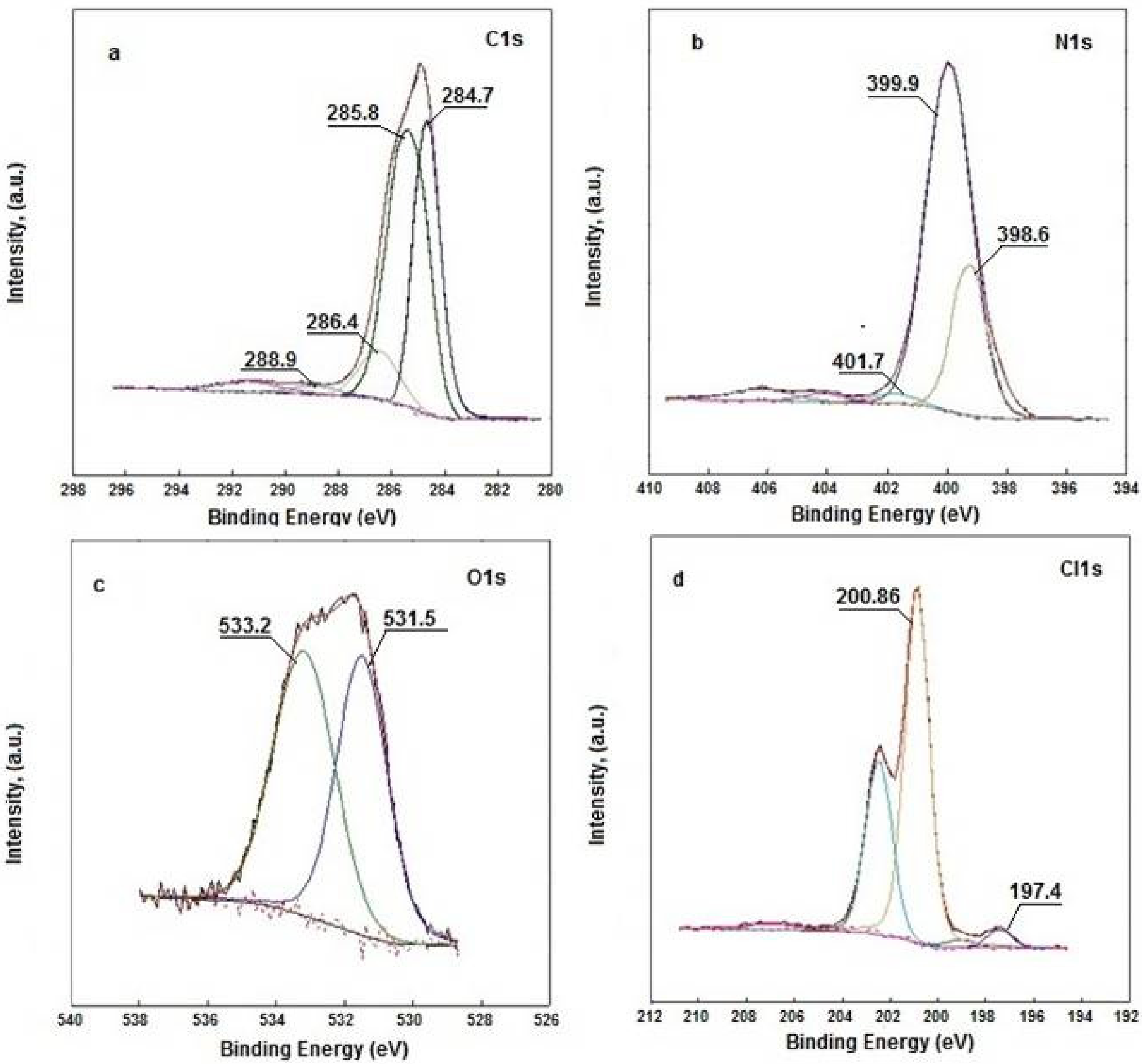

Figure S5. C1s (a) N1s (b), O1s (c) Cl1s (d) poly-DPDCB spectra (XPS).

Table S2. Approximation parameters of high-resolution O1s, Cl2p, N1s, C1s spectra (XPS).

Atom	DPDCB		poly-DPDCB	
	**Binding energy, E_b, eV**	**Intensity I, %**	**Binding energy, E_b, eV**	**Intensity I, %**
**O1s**	531.6	85.2	531.5	49.8
	533.3	14.8	533.2	50.2
**Cl2p**	200.86	87	200.86	94
	197.9	11	197.4	4
	206.9 (Satellite)	2	206.9 (Satellite)	2
**N1s**	-	-	398.6	23.1
	399.9	86	399.9	69.9
	401.65	12	401.7	2
	-	-	404.1 (Satellite)	2
	406.0 (Satellite)	2	406.0 (Satellite)	3
**C1s**	284.7	43	284.7	32.1
	285.9	40.4	285.8	44.0
	286.5	3	286.4	16.2
	288.9	10.5	288.9	5.1
	291.3 (Satellite)	3	291.3 (Satellite)	2.5

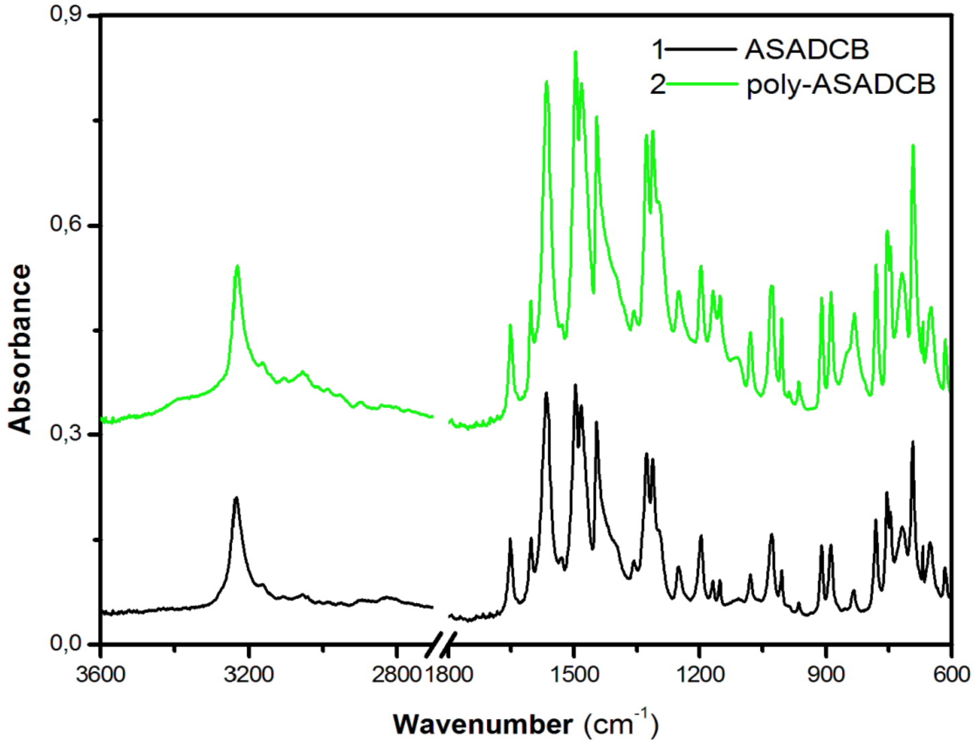

Figure S6. Comparison of ASADCB (1) and poly-ASADCB (2) FTIR spectra.
